# Clinical Outcomes of Breast-Involved Diffuse Large B-Cell Lymphoma Treated with R-CHOP: A Real-World Study with Insights into CNS Prophylaxis

**DOI:** 10.3390/curroncol33080493

**Published:** 2026-08-20

**Authors:** Thi Thu Huong Nguyen, Thi Yen Le, Thanh Tung Nguyen, Thanh Long Nguyen, Xuan Dai Nguyen, Tuan Anh Pham, Anh Tu Do, Thi Thanh Ha Lai, Van Quang Le

**Affiliations:** 1 Department of Oncology, Hanoi Medical University, Hanoi 100000, Vietnam; nguyenthu_huong@hmu.edu.vn (T.T.H.N.); nguyenxuandai99.hmu@gmail.com (X.D.N.); lequang@hmu.edu.vn (V.Q.L.); 2 Department of Quan Su Medical Oncology, Vietnam National Cancer Hospital, Hanoi 100000, Vietnam; 3 Department of Medical Oncology No. 7, Vietnam National Cancer Hospital, Hanoi 100000, Vietnam; leonguyenthanhtung@gmail.com; 4 Department of Medical Oncology No. 6, Vietnam National Cancer Hospital, Hanoi 100000, Vietnam; longnguyen.hmu@gmail.com; 5 Department A, Vietnam National Cancer Hospital, Hanoi 100000, Vietnam; phamtuananh@hmu.edu.vn; 6 Department of Tam Hiep Medical Oncology, Vietnam National Cancer Hospital, Hanoi 100000, Vietnam; doanhtu@bvk.org.vn; 7 Department of Medical Oncology, Thanh Hoa Oncology Hospital, Thanh Hoa 440000, Vietnam; laithanhha93.hmu@gmail.com

**Keywords:** diffuse large B-cell lymphoma, breast neoplasms, central nervous system neoplasms, methotrexate, R-CHOP, real-world evidence

## Abstract

This study evaluated clinical characteristics, treatment outcomes, and CNS relapse patterns in patients with breast-involved diffuse large B-cell lymphoma (DLBCL), a rare extranodal presentation with limited real-world data. We conducted a retrospective study on 33 consecutive patients with newly diagnosed breast-involved DLBCL treated from 2019 to 2024. All patients received R-CHOP. Baseline CNS screening—including neurological examination, fundoscopy, brain magnetic resonance imaging (MRI), and cerebrospinal fluid (CSF) analysis—was routinely performed. High-dose methotrexate (HD-MTX) was offered as CNS prophylaxis after completion of systemic therapy based on multidisciplinary team evaluation and clinician–patient shared decision-making according to institutional treatment protocols. Median age was 52.6 years; 84.8% had ECOG 0. Non-GCB subtype predominated (84.8%), and Ki-67 >70% was present in 69.7%. The overall response rate was 90.9%, with 84.8% complete responses. At a median follow-up of 44 months, 5-year Overall Survival (OS) and Progression-Free Survival (PFS) were 84.8% and 66.7%. CNS relapse occurred in 4 of 6 patients (66.7%) without prophylaxis, all within 5–11 months after R-CHOP, whereas no CNS relapses were observed among prophylaxis recipients (*p* < 0.001), although this observation should be interpreted with extreme caution given the very small non-prophylaxis subgroup (*n* = 6), limited statistical power, and non-randomized treatment allocation. Exploratory analyses suggested that bulky disease was associated with inferior OS. R-CHOP achieved high response rates and favorable long-term outcomes in breast-involved DLBCL. The absence of CNS relapse among HD-MTX prophylaxis recipients, contrasted with a high relapse rate in those without prophylaxis, provides only a hypothesis-generating observation that requires confirmation in larger prospective studies; this warrants further investigation of the role of systemic CNS prophylaxis.

## Simple Summary

Breast-involved diffuse large B-cell lymphoma (DLBCL) is a rare form of lymphoma, and there is limited real-world evidence to guide treatment, particularly regarding the risk of spread to the central nervous system (CNS). In this study, we analyzed 33 patients treated with standard R-CHOP therapy at a single center. We observed high response rates and favorable long-term survival outcomes. However, CNS relapse occurred frequently in patients who did not receive preventive treatment, while no CNS relapses were seen in those who received high-dose methotrexate (HD-MTX) as prophylaxis. These findings suggest a potential benefit of CNS prophylaxis in this high-risk population. Larger studies are needed to confirm these results and help guide treatment decisions.

## 1. Introduction

Diffuse large B-cell lymphoma (DLBCL) involving the breast is a rare clinical entity, accounting for approximately 1% of all non-Hodgkin lymphomas and less than 0.5% of all breast malignancies [1,2,3,4,5]. Owing to its rarity, historical treatment strategies have varied widely, ranging from surgery to combined-modality therapy. With the advent of systemic immunochemotherapy, R-CHOP has become the standard of care, making aggressive surgical approaches such as mastectomy largely unnecessary [1,6,7,8].

Despite the established role of R-CHOP in DLBCL, several management questions remain unresolved in breast-involved cases. One area of ongoing debate is the role of consolidative radiotherapy (RT), particularly for limited-stage disease [1,4]. Another important but controversial aspect is CNS prophylaxis. Although multiple international studies have suggested that systemic high-dose methotrexate (HD-MTX) may reduce CNS relapse, the overall quality of evidence remains limited due to the small number of cases and the lack of real-world data. In Southeast Asia, published reports on breast-involved DLBCL are scarce, and practice patterns—especially regarding CNS prophylaxis and RT—vary substantially across institutions [9,10,11,12,13].

Given these gaps, we conducted a retrospective study to describe the clinical characteristics, treatment patterns, and outcomes of patients with breast-involved DLBCL treated with R-CHOP at our center. In particular, we aimed to explore CNS relapse patterns and to evaluate the potential impact of systemic HD-MTX prophylaxis in routine clinical practice. Because CNS prophylaxis was offered based on clinician–patient discussion rather than randomized assignment, our analysis focuses on describing real-world treatment decisions and identifying signals that may help refine future management strategies.

## 2. Materials and Methods

### 2.1. Study Design and Participants

This was a retrospective, single-center observational cohort study. Between January 2019 and June 2024, all consecutive patients with newly diagnosed breast-involved DLBCL who met the predefined eligibility criteria and received first-line R-CHOP-based immunochemotherapy at our institution were included in this retrospective observational cohort. A total of 33 eligible patients were identified during the study period. The study protocol was reviewed and approved by the Institutional Ethics Committee of the Vietnam National Cancer Hospital (Approval No. 1582/QĐ-BVK). The study was conducted in accordance with the Helsinki Declaration of 1975, as revised in 2024. The reporting of this study conforms to STROBE guidelines [14]. Because this was a retrospective study based on routinely collected clinical data, no patients were prospectively recruited, and therefore no eligible patients declined participation.

The cohort included both primary breast lymphoma (PBL) and secondary breast lymphoma (SBL), classified according to the criteria proposed by Wiseman and Liao in 1972. PBL was defined as lymphoma confined to the breast, with or without regional nodal involvement, whereas SBL referred to breast involvement as part of systemic disease at initial presentation.

Eligible patients were ≥16 years of age, had a histopathologically confirmed diagnosis of DLBCL with breast involvement, an Eastern Cooperative Oncology Group (ECOG) performance status of 0–3, measurable disease per the Lugano classification, and adequate organ function. Patients with other lymphoma subtypes, prior malignancies, or uncontrolled severe comorbidities were excluded.

### 2.2. Interventions and Assessments

#### 2.2.1. Systemic Therapy

All patients received up to six 21-day cycles of the R-CHOP regimen, consisting of rituximab (375 mg/m^2^), cyclophosphamide (750 mg/m^2^), doxorubicin (50 mg/m^2^), and vincristine (1.4 mg/m^2^, capped at 2 mg) intravenously on Day 1, together with oral prednisolone (100 mg daily) on Days 1–5.

#### 2.2.2. Pathology and Immunophenotyping

Histopathological diagnosis was made by experienced hematopathologists. Immunohistochemical evaluation included CD20, CD3, CD10, BCL6, BCL2, MUM1, and Ki-67. Cell-of-origin classification was determined using the Hans algorithm. A Ki-67 index > 70% was documented as high proliferative activity. Fluorescence in situ hybridization (FISH) for MYC, BCL2, and BCL6 rearrangements was not routinely available during the study period.

#### 2.2.3. Imaging, Staging, and Response Assessment

Baseline staging was performed using Positron Emission Tomography/Computed Tomography (PET/CT) or contrast-enhanced Computed Tomography (CT) according to institutional availability. At our institution, comprehensive baseline CNS screening is part of the standard diagnostic evaluation for all newly diagnosed patients with breast-involved DLBCL. Accordingly, all consecutive eligible patients included in this retrospective cohort underwent the same standardized CNS assessment prior to treatment initiation, including neurological examination, fundoscopy, brain MRI, and cerebrospinal fluid analysis.

Treatment response was evaluated according to the Lugano 2014 criteria. Follow-up imaging was performed at the end of systemic therapy and subsequently at clinically appropriate intervals during surveillance.

#### 2.2.4. Radiotherapy

All cases were reviewed in a multidisciplinary team (MDT) setting involving hematology, medical oncology, radiation oncology, radiology, and pathology. Consolidative radiotherapy (30–50 Gy) was considered based on clinical features, including bulky disease at presentation, residual lesions on post-treatment imaging, or limited-stage disease, in accordance with institutional practice. Before radiotherapy initiation, all patients were counseled on potential benefits—including improved local control—and risks such as acute toxicity, lymphedema, or secondary malignancies. Radiotherapy was delivered only after informed patient consent.

#### 2.2.5. CNS Prophylaxis

High-dose intravenous methotrexate (HD-MTX, 3 g/m^2^) was administered over 3 h for 2–3 cycles as CNS prophylaxis after completion of R-CHOP. Before HD-MTX initiation, all patients underwent end-of-treatment response assessment using PET/CT or contrast-enhanced CT according to institutional availability. HD-MTX was initiated approximately 4–6 weeks after the last R-CHOP cycle in eligible patients who had adequately recovered hematologic and renal function. Supportive measures, including vigorous hydration, urine alkalinization, and leucovorin rescue, were administered according to institutional protocols. All cases were reviewed within a multidisciplinary team (MDT) setting, and decisions were made according to institutional treatment protocols, including assessment of CNS relapse risk using the CNS-IPI score, patient clinical status (particularly renal function), and anticipated treatment-related toxicity.

All patients received standardized counseling regarding the potential risks and benefits of CNS prophylaxis, and written informed consent was obtained prior to treatment. The final decision was individualized through clinician–patient shared decision-making within this structured framework. Supportive measures, including hydration, alkalinization, and leucovorin rescue, were administered per institutional protocols.

Because CNS prophylaxis was not randomly assigned but determined through multidisciplinary team discussion and clinician–patient shared decision-making according to institutional practice, treatment allocation may have been influenced by physician judgment and patient-related clinical factors. Consequently, confounding by indication and selection bias cannot be excluded. When HD-MTX could not be administered because of exceptional circumstances (e.g., treatment disruption during the COVID-19 pandemic) or patient refusal, patients remained in the non-prophylaxis group.

#### 2.2.6. Data Collection and Endpoints

Clinical data were collected from medical records, imaging studies, and pathology reports. Baseline demographic, clinical, laboratory, and histopathological characteristics were documented at diagnosis, with treatment-related and follow-up information updated at scheduled visits or through telephone contact.

The primary endpoint was overall survival (OS), defined as the time from diagnosis to death from any cause or last follow-up. Secondary endpoints included (1) progression-free survival (PFS)—time from diagnosis to disease progression, relapse, or death; (2) incidence of CNS relapse, stratified by prophylaxis status; (3) overall response rate (ORR) according to Lugano 2014 criteria; and (4) treatment-related toxicities, graded per CTCAE version 4.0. Exploratory endpoints included identifying potential prognostic factors for OS and PFS using Cox regression.

### 2.3. Statistical Analysis

All analyses were performed using SPSS version 20.0 (IBM Corp., Armonk, NY, USA). Baseline characteristics were summarized using descriptive statistics. Continuous variables were expressed as means with standard deviations or medians with ranges; categorical variables were presented as counts and percentages.

OS and PFS were estimated using the Kaplan–Meier method and compared across subgroups using the log-rank test. Univariate Cox proportional hazards regression analyses were conducted to explore potential prognostic factors for OS and PFS, including age, LDH, B symptoms, breast lesion, bulky disease, radiotherapy, and CNS prophylaxis. Given the limited number of progression and death events, Cox proportional hazards regression analyses were considered exploratory and were performed primarily to identify potential prognostic signals rather than establish independent prognostic factors. The definition of bulky disease (≥7.5 cm) was based on NCCN guideline recommendations [7]. Hazard ratios (HRs) with 95% confidence intervals (CIs) were reported.

The association between CNS prophylaxis and CNS relapse was assessed using Fisher’s exact test. All statistical tests were two-sided, and *p* < 0.05 was considered statistically significant.

## 3. Results

### 3.1. Patient and Disease Characteristics

A total of 33 patients with breast-involved DLBCL were enrolled between January 2019 and June 2024 (Table 1). The mean age was 52.6 ± 13.0 years (range: 18–80), and the majority presented with good performance status (ECOG 0 in 84.8%).

Breast involvement was unilateral in 78.8% and bilateral in 21.2% of patients. Bulky disease (≥7.5 cm) was documented in 21.2%. Based on the Wiseman–Liao criteria, 54.5% of cases were classified as primary breast lymphoma, while 45.5% had secondary breast involvement. According to the NCCN-IPI, most patients fell into the low–intermediate risk category (54.5%). Although these differences were not statistically significant, the numerical imbalance suggests that patients who did not receive CNS prophylaxis may have had less favorable baseline characteristics, which could have contributed to the observed outcomes through confounding by indication.

The distribution of CNS-IPI risk categories was similar between patients who received CNS prophylaxis and those who did not (*p* = 0.581). Most patients were classified as low or intermediate risk, with only a small proportion in the high-risk group.

Histopathologically, the non-germinal center B-cell (non-GCB) subtype predominated (84.8%). A high Ki-67 proliferation index (>70%) was found in 69.7%. Laboratory abnormalities included anemia (27.3%), elevated LDH (42.4%), and elevated β2-microglobulin (45.5%). Chronic HBV infection was recorded in 21.2% of patients.

Additional exploratory analyses comparing primary breast lymphoma (PBL) and secondary breast lymphoma (SBL) did not demonstrate statistically significant differences in baseline biological characteristics, including cell of origin and Ki-67 proliferation index, although some numerical variations were observed (Table 2).

### 3.2. Treatment Modalities, Clinical Outcomes, and CNS Relapse

All patients received R-CHOP immunochemotherapy. Consolidative radiotherapy was delivered to 12 patients (36.4%). CNS prophylaxis with high-dose methotrexate (HD-MTX) was administered to 27 patients (81.8%). Six patients (18.2%) did not receive HD-MTX prophylaxis. Five patients were unable to receive HD-MTX because treatment was disrupted during the COVID-19 pandemic owing to mandatory quarantine and restrictions on hospital access, while one patient declined prophylaxis after counseling. Six patients (18.2%) underwent unplanned breast surgery due to initial misdiagnosis as carcinoma (Table 3).

The overall response rate (ORR) was 90.9%, including 84.8% complete response (CR) and 6.1% partial response (PR). Three patients (9.1%) exhibited progressive disease (PD) during or after treatment.

**Figure 1:**
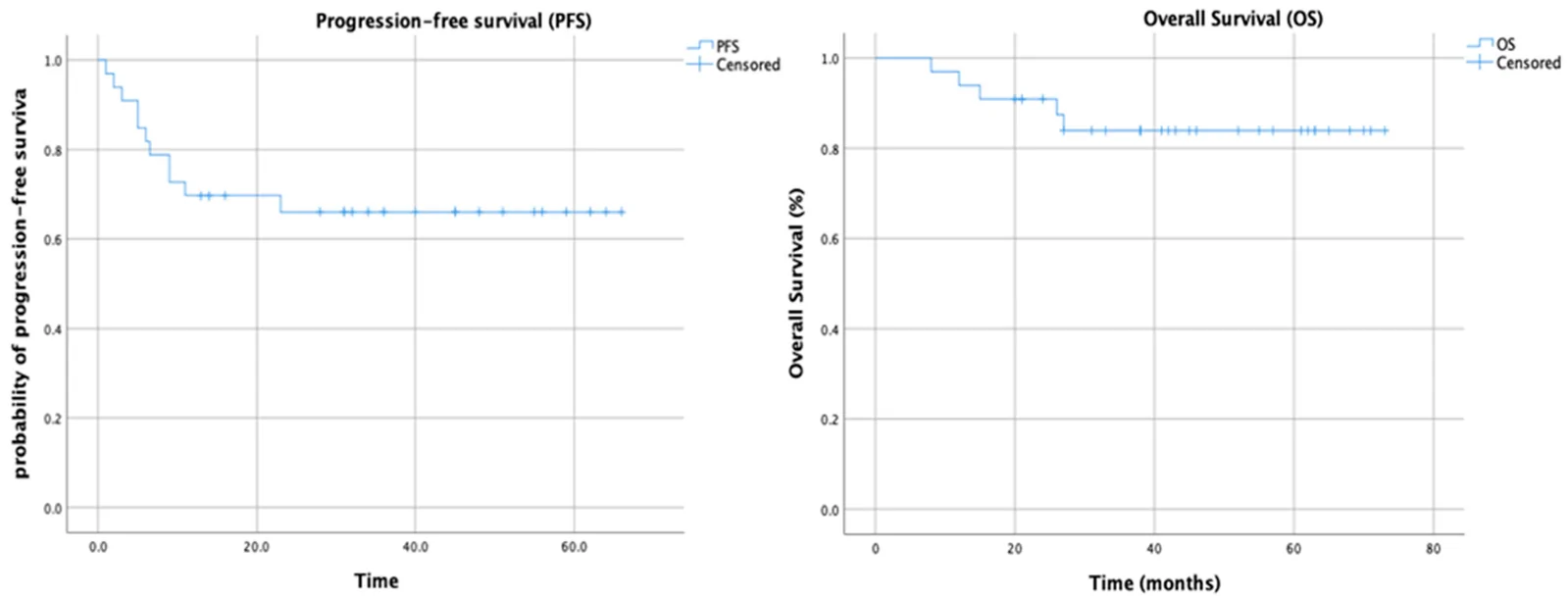
Progression-free survival (PFS) and overall survival (OS).

At a median follow-up of 44 months (range: 8–76), 28 patients (84.8%) were alive. Eleven relapse or progression events (33.3%) and five deaths (15.2%) were recorded during the study period. The estimated 5-year overall survival (OS) rate was 84.8%, and the 5-year progression-free survival (PFS) rate was 66.7% (Figure 1).

Four CNS relapse events were documented (12.1% of the cohort). All occurred among the six patients who did not receive HD-MTX prophylaxis (66.7%), whereas no CNS relapse was observed among prophylaxis recipients (0/27) (*p* < 0.001). Relapses occurred early, between 5 and 11 months after completing R-CHOP.

### 3.3. Prognostic Factors of Progression-Free Survival (PFS) and Overall Survival (OS)

In the exploratory univariate and multivariate Cox regression analysis, none of the assessed variables—including LDH elevation, age, bulky disease, B symptoms, breast laterality, comorbidities, and consolidative radiotherapy—reached statistical significance for PFS (Table 4 and Table 5). For OS, bulky disease was associated with inferior survival. Exploratory analysis did not demonstrate a statistically significant association between cell of origin (GCB vs. non-GCB) and clinical outcomes, including overall survival, progression-free survival, or CNS relapse. Given the limited number of progression and death events, these analyses should be interpreted cautiously and were not intended to establish definitive prognostic associations.

### 3.4. Safety and Tolerability

Across 192 cycles of R-CHOP, grade 3–4 neutropenia occurred in 7.8% of cycles (including 2.1% grade 4; 75% of grade 4 episodes were febrile). Grade 3 anemia occurred in 1%, and no grade ≥ 3 thrombocytopenia was observed (Table 6). HD-MTX prophylaxis was associated with grade 3 neutropenia in 2.8% of cycles and no severe anemia or thrombocytopenia. Common non-hematologic toxicities included alopecia (100%), anorexia (63.6%), nausea/vomiting (36.4%), peripheral neuropathy (30.3%), and elevated liver enzymes (24.2%). Two episodes of renal dysfunction (6.1%) occurred during treatment, both of which resolved with supportive care.

## 4. Discussion

This retrospective cohort provides meaningful real-world insights into breast-involved DLBCL in Southeast Asia, a rare and clinically distinctive form of extranodal lymphoma [1,2,3]. Despite its low incidence, breast DLBCL presents important diagnostic and therapeutic challenges, and high-quality data remain scarce. Our cohort of 33 patients, uniformly treated with R-CHOP and systematically evaluated—with comprehensive CNS screening including fundoscopy, brain MRI, and CSF analysis—represents a valuable contribution to the existing literature.

With a median follow-up of 44 months, our findings reaffirm the effectiveness of R-CHOP in achieving durable disease control [9,15,16,17,18,19,20], reflected by an ORR of 90.9%, CR rate of 84.8%, and 5-year OS and PFS rates of 84.8% and 66.7%, respectively. These outcomes align closely with international series and support the continued use of standard immunochemotherapy even in the context of the predominantly non-GCB phenotype observed in our cohort (84.8%) [9,21]. The high proliferative index (Ki-67 > 70% in 69.7%) reinforces observations that breast-involved DLBCL may represent a biologically aggressive subtype similar to other immune-privileged-site lymphomas [21,22,23,24].

A notable biological feature of our cohort is the predominance of the non-GCB subtype (84.8%). Unlike MYC rearrangements, which are typically associated with the GCB subtype, this distribution suggests that alternative mechanisms may drive breast tropism. Emerging evidence indicates that epigenetic dysregulation plays a central role in DLBCL pathogenesis and may influence extranodal localization [25]. These processes may be particularly relevant in sites such as the breast; however, such interpretations remain hypothesis-driven and are not directly supported by the molecular data from the present study. Future studies integrating genomic and epigenomic profiling are warranted to better define the biology of breast-involved disease. In addition, a relatively high prevalence of chronic hepatitis B virus (HBV) infection (21.2%) was observed in our cohort. This finding is consistent with the epidemiological context of Vietnam, where HBsAg seroprevalence ranges from approximately 10% to 20% [26]. Given the risk of HBV reactivation with rituximab-containing regimens, systematic screening and antiviral prophylaxis are essential. In our cohort, both were applied uniformly according to institutional and national guidelines.

A notable strength of this study is the systematic evaluation of CNS prophylaxis. No CNS relapses occurred among the 27 patients who received high-dose methotrexate, whereas 4 of 6 patients, 66.7% of those without prophylaxis, developed CNS relapse within the first year. This finding, however, must be interpreted with caution. The non-prophylaxis group was small, and baseline clinical imbalances—including numerically higher rates of bilateral breast involvement, bulky disease, and secondary breast lymphoma—combined with non-randomized treatment allocation, may have influenced outcomes. This observation should therefore be regarded as hypothesis-generating rather than evidence of a causal protective effect. Importantly, most patients in the non-prophylaxis group did not forgo HD-MTX because of clinical contraindications but because treatment was disrupted during the COVID-19 pandemic owing to mandatory quarantine and restricted access to hospital services, while one patient declined prophylaxis after counseling. These exceptional circumstances should be considered when interpreting comparisons between the prophylaxis and non-prophylaxis groups. These exceptional circumstances should be considered when interpreting comparisons between the prophylaxis and non-prophylaxis groups. Consequently, the observed association between HD-MTX prophylaxis and CNS relapse should be regarded as hypothesis-generating rather than evidence of a causal protective effect. The timing of HD-MTX administration remains an area of ongoing debate. While intercalated HD-MTX may provide earlier CNS exposure, it may also increase toxicity and compromise R-CHOP dose intensity. In our cohort, HD-MTX was administered after completion of systemic therapy to preserve treatment feasibility in a real-world setting. The impact of timing on CNS relapse risk could not be evaluated and warrants further investigation.

The magnitude and early timing of relapse in the non-prophylaxis group are consistent with the known CNS tropism of breast-involved and other immune-privileged-site lymphomas. Nevertheless, given the non-randomized nature of prophylaxis decisions and the limited sample size, these results should be interpreted as hypothesis-generating and warrant validation in larger studies. Our findings should also be interpreted in the context of recent international evidence. A large multicenter study by Wilson et al. evaluating ultra-high-risk LBCL, including patients with breast involvement, did not demonstrate a significant reduction in CNS relapse with HD-MTX prophylaxis, highlighting the ongoing uncertainty regarding its clinical benefit despite current guideline recommendations. These findings reinforce that patient selection for CNS prophylaxis remains challenging and underscore the need for prospective studies in biologically high-risk extranodal DLBCL [27].

Toxicities associated with R-CHOP and HD-MTX prophylaxis were manageable. Severe neutropenia occurred in 7.8% of R-CHOP cycles, while HD-MTX resulted in only 2.8% grade 3 neutropenia without grade 4 hematologic events. Two cases of transient renal dysfunction were observed, both resolving with supportive care. These findings confirm that HD-MTX prophylaxis is feasible and generally well tolerated, even in resource-limited settings.

Exploratory Cox analysis identified bulky disease as a factor associated with inferior OS, consistent with global DLBCL prognostic models [15,16,17]. No variable predicted PFS, likely reflecting the limited number of progression events and the overall high response rates with frontline R-CHOP. Given the limited number of progression events and particularly only five deaths, multivariable Cox regression analyses were at risk of overfitting. Therefore, these findings should be regarded as preliminary, exploratory, and hypothesis-generating rather than definitive evidence of independent prognostic factors.

The role of consolidative radiotherapy (RT) in breast DLBCL remains debated [6,28]. In our study, radiotherapy was administered in 36.4% of patients based on clinical judgment. Exploratory analyses did not demonstrate a statistically significant survival benefit; however, these findings should be interpreted with caution due to the small sample size and non-standardized treatment selection. Local recurrence was observed among patients who did not receive consolidative radiotherapy; however, this descriptive observation does not establish a causal benefit because radiotherapy was administered according to clinical judgment rather than random allocation, and treated patients likely represented a selected subgroup with different baseline characteristics [6,11]. The role of consolidative RT therefore remains uncertain and warrants prospective evaluation.

The therapeutic landscape of diffuse large B-cell lymphoma (DLBCL) is evolving rapidly, particularly with the introduction of novel frontline regimens. The addition of the anti-CD79b antibody–drug conjugate Polatuzumab vedotin to R-CHP has demonstrated improved progression-free survival compared with standard R-CHOP in the POLARIX trial [29], establishing a new potential standard of care in selected populations. Such regimens may further enhance systemic disease control and could potentially influence the risk of CNS relapse, particularly in biologically aggressive or extranodal subtypes such as breast-involved DLBCL. Beyond polatuzumab-based frontline therapy, CD19-directed immunotherapies, including bispecific antibodies and CAR T-cell therapy, are rapidly reshaping the treatment landscape of DLBCL. Although currently reserved mainly for relapsed or refractory disease, these approaches may ultimately expand therapeutic options for biologically aggressive extranodal DLBCL [30]. However, the real-world applicability of these advances remains challenging in many low- and middle-income countries. In Vietnam, polatuzumab-based therapy is currently cost-prohibitive for the majority of patients, and R-CHOP remains the most widely used frontline regimen.

This study has several limitations. The modest sample size—particularly the small non—prophylaxis subgroup—may have limited statistical power. As a single-center study, findings may not be fully generalizable. CNS prophylaxis was not randomized, introducing potential selection bias. Furthermore, the limited number of progression and death events reduced the reliability of multivariable regression analyses and increased the risk of model overfitting. Consequently, these analyses should be interpreted as exploratory. Data on MYC translocations were unavailable; however, given that this alteration is typically associated with the GCB subtype and the majority of our cohort was non-GCB (84.8%), this gap is unlikely to substantially alter the overall conclusions. Counterbalancing these limitations are several methodological strengths: uniform R-CHOP treatment, systematic baseline CNS screening, consistent imaging assessment, and complete follow-up. In the context of a rare disease with limited global data, these results offer clinically relevant, real-world evidence [1,2,3,6,9,18,19,20,21,22,28].

Overall, our findings suggest that breast-involved DLBCL may warrant systematic consideration of CNS prophylaxis, particularly using systemic HD-MTX. The absence of CNS relapse among prophylaxis recipients, contrasted with a high relapse rate in non-recipients, underscores the need for further evaluation of CNS-directed strategies in this setting [10,11,12,13]. These results highlight the biological distinctiveness of breast lymphoma and support the rationale for future studies incorporating molecular profiling and multicenter collaboration to refine risk-adapted treatment approaches.

## 5. Conclusions

R-CHOP achieved high response rates and favorable long-term outcomes in breast-involved DLBCL. The absence of CNS relapse among HD-MTX prophylaxis recipients, contrasted with a high relapse rate in those without prophylaxis, provides a clinically relevant hypothesis-generating signal suggesting a potential role of systemic CNS prophylaxis that requires validation in larger studies.

## Figures and Tables

**Table 1:** Baseline Patient and Disease Characteristics.

Characteristic	Value/*n* (%)	CNS Prophylaxis		*p*-Value
		Yes	No	
Total Patients	33 (100)	27 (81.8)	6 (18.2)	-
Age, years				
Mean ± SD	52.6 ± 13.0	52.2 ± 13.7	54.7 ± 10.4	0.624 ^#^
Median (Range)	53 (18–80)	52 (18–80)	53 (34–69)	-
ECOG Performance Status				
0	28 (84.8)	23 (85.2)	5 (83.3)	0.216 *
1–3	5 (15.2)	4 (14.8)	1 (16.7)	
B Symptoms present	8 (24.2)	7 (25.9)	1 (16.7)	0.616 *
Tumor side in breast				
Right	16 (48.5)	14 (51.9)	2 (33.3)	0.289 *
Left	10 (30.3)	9 (33.3)	1 (16.7)	
Bilateral	7 (21.2)	4 (14.8)	3 (50.0)	
Bulky disease ≥ 7.5 cm	7 (21.2)	4 (14.8)	3 (50.0)	0.093 *
Ann Arbor Classification				0.375 * (PBL vs. SBL)
Primary Breast Lymphoma (PBL)	18 (54.5)	16 (59.3)	2 (33.3)	
Stage IE	6	5	1	0.524 *
Stage IIE	11	10	1	
Stage IV	1	1	0	
Secondary Breast Lymphoma (SBL)	15 (45.5)	11 (40.7)	4 (66.7)	
NCCN-IPI Risk Group				
Low	13 (39.4)	11 (40.7)	2 (33.3)	0.874 *
Low–Intermediate	18 (54.5)	15 (55.6)	3 (50.0)	
High–Intermediate	2 (6.1)	1 (3.7)	1 (16.7)	
CNS-IPI Risk Group				
Low	17 (51.5)	15 (55.6)	2 (33.3)	0.581 *
Intermediate	14 (42.4)	10 (37.0)	4 (66.7)	
High	2 (6.1)	2 (7.4)	0	
Histologic subtype				
GCB	5 (15.2)	3 (11.1)	2 (33.3)	0.464 *
Non-GCB	28 (84.8)	24 (88.9)	4 (66.7)	
Ki-67 > 70%	23 (69.7)	18 (66.7)	5 (83.3)	0.640 *
Laboratory findings at Diagnosis				
Anemia	9 (27.3)	9 (33.3)	0	1.000 *
Elevated LDH	14 (42.4)	11 (40.7)	3 (50.0)	1.000 *
Elevated ß2-microglobulin	15 (45.5)	10 (37.5)	5 (83.3)	0.085 *
Hepatitis B Virus Positive	7 (21.2)	6 (22.2)	1 (16.7)	1.000 *

Abbreviations: ECOG, Eastern Cooperative Oncology Group; NCCN-IPI, National Comprehensive Cancer Network International Prognostic Index; LDH, lactate dehydrogenase; ^#^ Independent Samples *t*-test; * Fisher’s Exact Test.

**Table 2:** Comparison of baseline biological characteristics between primary breast lymphoma (PBL) and secondary breast lymphoma (SBL).

Characteristic	Value/*n* (%)	PBL vs. SBL		*p*-Value
		PBL	SBL	
Total Patients	33 (100)	18 (54.5)	15 (45.5)	-
Histologic subtype				
GCB	5 (15.2)	2 (11.1)	3 (20.0)	0.639 *
Non-GCB	28 (84.8)	16 (88.9)	12 (80.0)	
Ki-67 > 70%	23 (69.7)	13 (72.2)	10 (66.7)	1.000

* Fisher’s Exact Test.

**Table 3:** Treatment Modalities, Clinical Outcomes, and CNS Relapse.

Parameter	*n* (%)
Treatment Modality	
R-CHOP Chemotherapy	33 (100)
Followed by Radiotherapy	12 (36.4)
CNS Prophylaxis	27 (81.8)
Unplanned Surgery *	6 (18.2)
Response After All Treatment	
Overall Response Rate (ORR)	30 (90.9)
Complete Response (CR)	28 (84.8)
Partial Response (PR)	2 (6.1)
Progressive Disease (PD)	3 (9.1)
Follow-up Status	
Median Follow-up, months (range)	44 (8–76)
Alive at Last Follow-up	28 (84.8)
Relapse events	11 (33.3)
Death events	5 (15.2)
Estimated 5-year Overall Survival (OS)	84.8%
Estimated 5-year Progression-Free Survival (PFS)	66.7%
CNS Relapse	
Total CNS relapse events	4/33 (12.1 (95% CI 3.0–24.2))
CNS relapse in patients with prophylaxis	0/27 (0)
CNS relapse in patients without prophylaxis	4/6 (66.7)
*p*-value (Fisher’s Exact Test) **	<0.001
Timing of CNS relapse after R-CHOP	5, 6.5, 9, and 11 months

* Six patients underwent surgery due to an initial misdiagnosis of breast adenocarcinoma; final pathology confirmed lymphoma. ** *p*-value calculated using Fisher’s Exact Test comparing relapse rates between prophylaxis and non-prophylaxis groups.

**Table 4:** Univariate and Multivariate Cox Regression Analysis—Prognostic Factors for Progression-Free Survival (PFS).

Prognostic Factor *	Univariate			Multivariate		
	HR	95% CI	*p*-Value	HR	95% CI	*p*-Value
LDH (≤ULN vs. >ULN)	1.25	0.38–4.10	0.716	1.560	0.208–11.677	0.665
Age (≤60 vs. >60)	0.86	0.23–3.25	0.823	1.600	0.285–8.995	0.594
Consolidation radiotherapy (yes vs. no)	0.31	0.07–1.42	0.130	0.247	0.031–1.936	0.183
Bulky disease (no vs. yes)	0.59	0.16–2.24	0.440	0.407	0.058–2.834	0.364
B symptoms (no vs. yes)	1.32	0.35–4.97	0.686	0.370	0.037–3.702	0.398
Breast lesion (unilateral vs. bilateral)	0.37	0.11–1.25	0.109	0.267	0.034–2.089	0.208
Comorbidities † (no vs. yes)	0.96	0.28–3.27	0.945	1.075	0.240–4.823	0.925
CNS relapsed (no vs. yes)	0.11	0.03–0.45	0.002	0.157	0.030–0.811	0.027
Primary vs. Secondary breast lymphoma	0.95	0.289–3.104	0.928	4.423	0.653–29.947	0.128

*p*-values derived from univariate and multivariate Cox proportional hazards models. † Comorbidities include diabetes, hypertension, hepatitis B, diabetes insipidus, and bronchial asthma. * The latter group served as the reference group.

**Table 5:** Univariate and Multivariate Cox Regression Analysis—Prognostic Factors for Overall Survival (OS).

Prognostic Factor *	Univariate			Multivariate		
	HR	95% CI	*p*-Value	HR	95% CI	*p*-Value
LDH (≤ULN vs. >ULN)	0.89	0.15–5.34	0.899	0.003	0.000–1.456	0.065
Age (≤60 vs. >60)	0.57	0.06–5.08	0.612	0.334	0.008–14.364	0.568
Consolidation radiotherapy (yes vs. no)	0.38	0.04–3.42	0.389	0.261	0.008–8.294	0.447
Bulky disease (no vs. yes)	0.14	0.02–0.82	0.030	0.016	0.000–0.688	0.031
B symptoms (no vs. yes)	2.28	0.38–13.65	0.367	0.588	0.018–18.801	0.764
Breast lesion (unilateral vs. bilateral)	0.36	0.06–2.14	0.261	0.057	0.001–5.696	0.223
Comorbidities † (no vs. yes)	1.12	0.19–6.68	0.905	0.625	0.034–11.540	0.752
CNS relapsed (no vs. yes)	0.18	0.03–1.12	0.067	0.445	0.043–4.612	0.497
Primary vs. Secondary breast lymphoma	0.49	0.082–2.941	0.435	0.057	0.001–3.039	0.158

*p*-values derived from univariate and multivariate Cox proportional hazards models. † Comorbidities include diabetes, hypertension, hepatitis B, diabetes insipidus, and bronchial asthma. * The latter group served as the reference group.

**Table 6:** Treatment-Related Toxicities During R-CHOP and CNS Prophylaxis.

Toxicity Type	R-CHOP Regimen (*n* = 192 Cycles)	High-Dose MTX Prophylaxis (*n* = 71 Cycles)
Hematologic Toxicity (Grade 3–4)		
Neutropenia	7.8% (Grade 3: 5.7%, Grade 4: 2.1%). 75% of Grade 4 cases were febrile.	2.8% (Grade 3 only)
Anemia	1.0% (Grade 3 only)	0%
Thrombocytopenia	0%	0%
Non-Hematologic toxicities (Any Grade, *n* = 33 patients) ^1^		
Alopecia	100%	-
Anorexia	63.6%	-
Nausea/Vomiting	36.4%	-
Peripheral Neuropathy	30.3%	-
Elevated Liver Enzymes	24.2%	-
Renal Failure	6.1% (2 cases)	-

Percentages for hematologic toxicities are cycle-based, whereas non-hematologic toxicities are patient-based and were recorded across the entire treatment course. ^1^ Non-hematologic toxicities were recorded for the entire treatment course and were primarily associated with the R-CHOP regimen. Most of these events were Grade 1–2 in severity.

## Data Availability

The datasets generated during and/or analyzed during the current study are not publicly available but are available from the corresponding author on reasonable request.

## Abbreviations

The following abbreviations are used in this manuscript:

DLBCL	Diffuse Large B-Cell Lymphoma
CNS	Central Nervous System
HD-MTX	High-Dose Methotrexate
MRI	Magnetic Resonance Imaging
CSF	Cerebrospinal Fluid
ECOG	Eastern Cooperative Oncology Group
GCB	Germinal Center B-cell
non-GCB	Non-Germinal Center B-cell
OS	Overall Survival
PFS	Progression-Free Survival
ORR	Overall Response Rate
CR	Complete Response
PR	Partial Response
PD	Progressive Disease
RT	Radiotherapy
MDT	Multidisciplinary Team
PET/CT	Positron Emission Tomography/Computed Tomography
CT	Computed Tomography
FISH	Fluorescence In Situ Hybridization
PBL	Primary Breast Lymphoma
SBL	Secondary Breast Lymphoma
HBV	Hepatitis B Virus
CTCAE	Common Terminology Criteria for Adverse Events
CI	Confidence Interval
HR	Hazard Ratio
